# Curvature of gastrocnemius muscle fascicles as function of muscle–tendon complex length and contraction in humans

**DOI:** 10.14814/phy2.15739

**Published:** 2023-06-03

**Authors:** Jule Heieis, Jonas Böcker, Olfa D'Angelo, Uwe Mittag, Kirsten Albracht, Eckhard Schönau, Andreas Meyer, Thomas Voigtmann, Jörn Rittweger

**Affiliations:** ^1^ Institute of Aerospace Medicine German Aerospace Center Cologne Germany; ^2^ Faculty of Medical Engineering and Technomathematics FH Aachen University of Applied Science Aachen Germany; ^3^ Institute of Materials Physics in Space German Aerospace Center Cologne Germany; ^4^ Children's Hospital University of Cologne Cologne Germany; ^5^ Present address: Institute for Multiscale Simulation Universität Erlangen‐Nürnberg Erlangen Germany

**Keywords:** biomechanics, connective tissue, physiology, ultrasound

## Abstract

It has been shown that muscle fascicle curvature increases with increasing contraction level and decreasing muscle–tendon complex length. The analyses were done with limited examination windows concerning contraction level, muscle–tendon complex length, and/or intramuscular position of ultrasound imaging. With this study we aimed to investigate the correlation between fascicle arching and contraction, muscle–tendon complex length and their associated architectural parameters in gastrocnemius muscles to develop hypotheses concerning the fundamental mechanism of fascicle curving. Twelve participants were tested in five different positions (90°/105°*, 90°/90°*, 135°/90°*, 170°/90°*, and 170°/75°*; *knee/ankle angle). They performed isometric contractions at four different contraction levels (5%, 25%, 50%, and 75% of maximum voluntary contraction) in each position. Panoramic ultrasound images of gastrocnemius muscles were collected at rest and during constant contraction. Aponeuroses and fascicles were tracked in all ultrasound images and the parameters fascicle curvature, muscle–tendon complex strain, contraction level, pennation angle, fascicle length, fascicle strain, intramuscular position, sex and age group were analyzed by linear mixed effect models. Mean fascicle curvature of the medial gastrocnemius increased with contraction level (+5 m^−1^ from 0% to 100%; *p* = 0.006). Muscle–tendon complex length had no significant impact on mean fascicle curvature. Mean pennation angle (2.2 m^−1^ per 10°; *p* < 0.001), inverse mean fascicle length (20 m^−1^ per cm^−1^; *p* = 0.003), and mean fascicle strain (−0.07 m^−1^ per +10%; *p* = 0.004) correlated with mean fascicle curvature. Evidence has also been found for intermuscular, intramuscular, and sex‐specific intramuscular differences of fascicle curving. Pennation angle and the inverse fascicle length show the highest predictive capacities for fascicle curving. Due to the strong correlations between pennation angle and fascicle curvature and the intramuscular pattern of curving we suggest for future studies to examine correlations between fascicle curvature and intramuscular fluid pressure.

## Graphical abstract

With this study we were able to describe fascicle curvature as detailed as it has not been done before. Strong correlations have been found between concave fascicle curvature towards the deep aponeurosis and the deep pennation angle and the inverse fascicle length. There has also been found evidence for intramuscular differences in fascicle curvature in a central‐peripheral pattern.

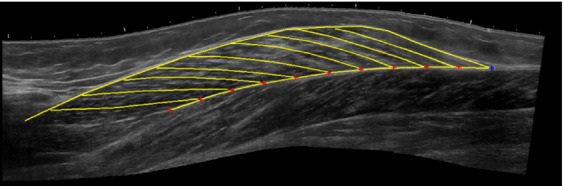

## 1 INTRODUCTION

Many muscles in humans are pennated, which means that the muscle's microscopic functional units (i.e., the sarcomeres) as well as its macroscopic functional units (i.e., the fascicles) are contracting at an oblique angle with the aponeuroses and tendon. It has been hypothesized that fascicle pennation diminishes force transfer toward the aponeurosis and tendon which scales with the cosine of pennation angle *α*, that is, with the fascicle's projection onto the aponeurosis (Zajac, 1989).

This has obvious implications on the mechanical behavior of the entire muscle construct, and it has been demonstrated that pennation angle, in addition to muscle length and muscle cross‐section, is an important factor in designing functional anatomical muscles (Lieber & Fridén, 2000).

In this reasoning, the muscle fascicles are treated as straight lines. However, it is known for more than two decades that fascicles can assume a curved path (Maganaris et al., 1998; van Leeuwen & Spoor, 1992). Thus, Muramatsu et al. (2002) have investigated fascicle curvature in the medial gastrocnemius with ultrasound imaging, and reported a direct relationship between fascicle curvature on one hand, and contraction level and degree of plantarflexion on the other hand. Subsequent studies have confirmed these findings (Namburete & Wakeling, 2012; Wang et al., 2009). Two‐dimensional (2D) ultrasound imaging over the entire length of the medial and lateral gastrocnemius muscles revealed intramuscular and intrafascicular variations of fascicle curvature, where the curvature was most pronounced in proximal and superficial regions (Namburete & Wakeling, 2012). However, (Namburete & Wakeling, 2012) measured only in mid‐belly regions, and did not assess the effects of sub‐maximal contraction levels. Wick et al. (2018) studied the differences in soleus muscle architecture from when it is isolated to when it is packed within the calf (Wick et al., 2018). Interestingly, the fascicle curvature increased, when packed, assuming an interaction effect between adjacent muscles. When fascicle curvature is transferred to the third dimension in an in silico model that assumed fascicles to be arranged along longitudinal curved sheets, it was suggested that the 3D fascicle projection onto the 2D image plane could explain the observed variation in 2D fascicle curvature (Rana et al., 2014). For the soleus and biceps brachii muscles 3D models have been generated from experimental anatomical and biomechanical data (Blemker et al., 2005; Seydewitz et al., 2019). Based on the experimental data some fascicles had a curved path. Modeling the muscles this way enabled the model to reproduce experimental findings like nonuniform strains within the muscle (Blemker et al., 2005).

Thus, although previous studies have revealed the occurrence of curved fascicles, the exact mechanisms on how curving emerges are still under debate. For example, both the studies by Muramatsu et al. (2002) and (Namburete & Wakeling, 2012) found that the muscle–tendon complex (MTC) length has an impact on curvature. However, the physical mechanism(s) through which MTC length could cause fascicle curvature remains unclear.

It is our hypothesis that any deviation from straightness in fascicles are evidence of intramuscular stresses misaligned with the fascicles' orientation. Hydrostatic pressure gradients could be one source of such unaligned stresses (Sejersted et al., 1984). The emergence of pressure gradients could be a consequence of volume consistency (Otten, 1988). However, this explanation thus remains hypothetical, and alternative explanations are available, as discussed in Section 4.

As mechanistic hypotheses must be based on experimental observations of intramuscular tissue mechanics, we collected a comprehensive database to model statistically the relationship between contraction‐related fascicle kinematics (including pennation angle), contraction levels, and fascicle curvature. Our guiding hypotheses are that contraction‐related fascicle curvature depends on muscle–tendon complex length and contraction level, and potentially also on pennation angle and fascicle length. In addition, we also consider possible effects of age and sex, because of the known impact of these parameters on muscle composition (Forsberg et al., 1991).

We consider in our experiments that within the plantar flexor muscles, the two gastrocnemius muscles actuate both knee and ankle joints, while the soleus muscle only operates the ankle joint. Accordingly, besides variation in ankle joint angle we included variation in knee joint angle, thereby introducing variation in MTC length of the gastrocnemius but not of the soleus muscle. Besides, we take advantage of panoramic views over the entire muscle length that recently became available, to study intra‐muscular variation of fascicle curvature.

## 2 MATERIALS AND METHODS

### 2.1 Study design

**FIGURE 1:**
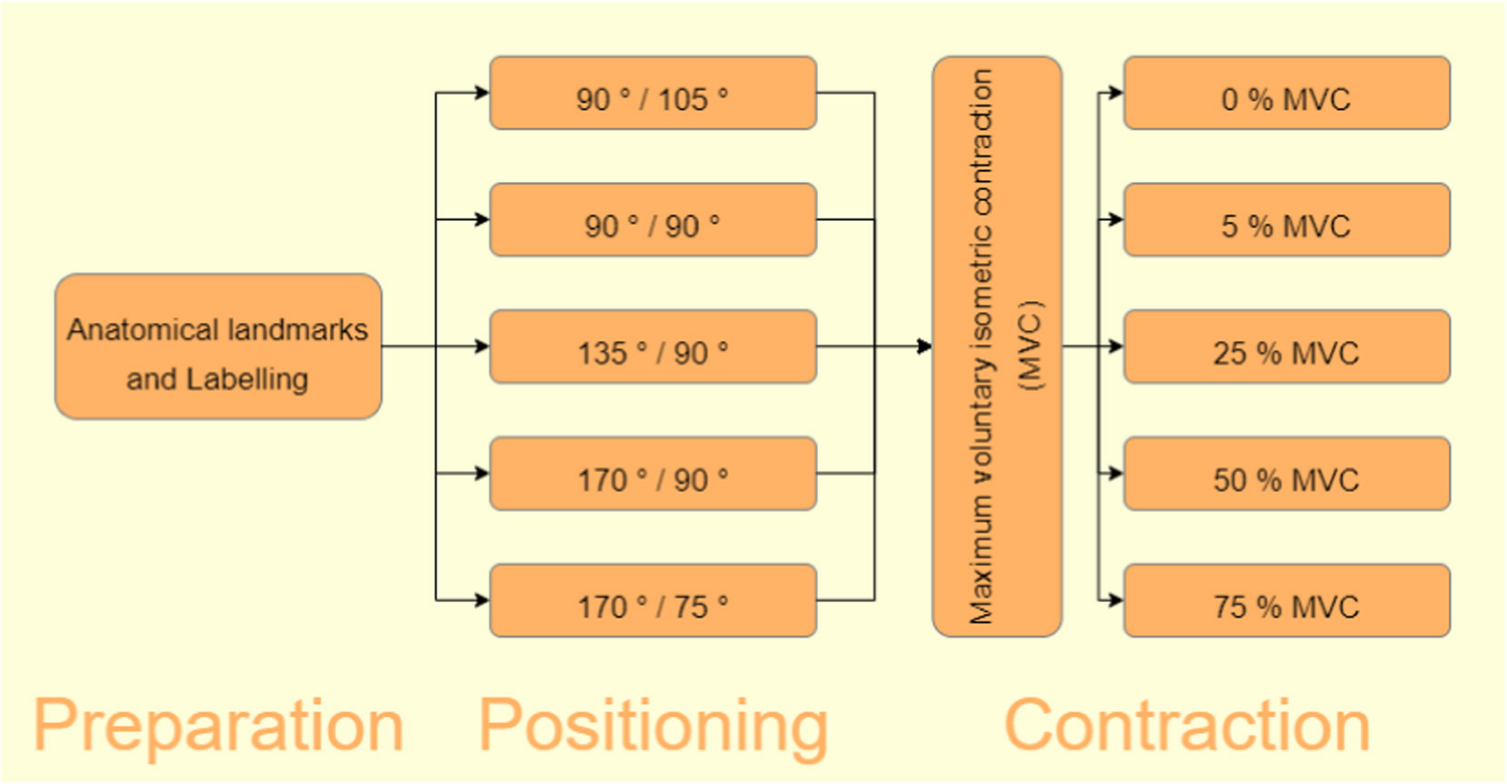
Study flow. Joint angles are given as geometrical knee/ankle angle.

The study was approved by the North Rhine Medical Association's ethics committee, and conducted following a crossover study design. Fascicle curvature of the gastrocnemius muscles of the right leg was investigated in five different positions, namely 90°/105°, 90°/90°, 135°/90°, 170°/90°, and 170°/75° (each reported as geometrical knee/ankle angle) and under the contraction states 0%, 5%, 25%, 50%, and 75% of maximum voluntary isometric contraction (MVC), as can be seen in Figure 1.

### 2.2 Participants

An a priori sample size estimation based on the findings of Muramatsu et al. revealed a sufficient sample size of 12 participants, achieving a power of 0.99 with a significance level of 0.05. Therefore, 12 healthy participants were included into the study. Inclusion criterion was defined as age either between 18 and 30 or between 60 and 70 years of age. The only exclusion criterion was the presence of injuries or diseases that could interfere with calf muscle contraction.

### 2.3 Data acquisition

Joint angles were recorded during the ultrasound scans via marker‐based motion capturing (Vicon Motion Systems Ltd). Therefore, participants were equipped with eight reflective markers, 14 mm in diameter, at significant anatomical landmarks of their right leg (listed in Table 1). The measurement volume's *x*‐axis was defined along the front edge of the seat, *y*‐axis along the side edge, and *z*‐axis vertically. The order of the positions was randomized. In each position the participants performed one assessment of MVC in which they performed a maximum contraction of plantarflexion supported by verbal encouragement. After the MVC followed graded isometric contractions at 5%, 25%, 50%, and 75% of MVC. The medial and lateral gastrocnemius were scanned along their medio‐lateral centerline using panoramic ultrasound imaging (LOGIQ S8 XDclear+, GE Healthcare) at rest and during constant isometric contractions. Before image acquisition the muscles' muscle–tendon junction (MTJ) and the transversal epimysial boundaries at mid‐belly were detected sonographically. The medio‐lateral centerline was then defined as straight line from MTJ through the mid distance between the transversal boundaries to the knee joint cleft. For panoramic ultrasound imaging the probe was hand guided slowly along a straight line in its longitudinal axis, perpendicularly oriented toward the skin surface. The scanning process took about 10 s per image. The ultrasound device stitched the collected images into a panoramic image. During scanning dynamometry (IsoMed 2000 dynamometer, D&R Ferstl GmbH) measured the plantarflexion torque during image acquisition in order to quantify the muscle's contraction state. The dynamometer's axis of rotation was aligned with the lateral malleolus.

**TABLE 1:** Marker set for joint angle recording.

Anatomical landmark	Abbreviation	Body segment
Greater trochanter	TROC	Proximal thigh
Lateral epicondyle	LEPI	Distal thigh
Fibular head	FIB	Proximal shank
Lateral malleolus	LMAL	Distal shank
Achilles tendon insertion	ATI	Proximal foot
Second metatarsus^a^	TOE	Distal foot
Medial epicondyle	MEPI	Backup for thigh reconstruction
Medial malleolus	MMAL	Backup for shank reconstruction

^a^ At point where the distance to the sole is the same distance between sole and ATI.

### 2.4 Data processing

**FIGURE 2:**
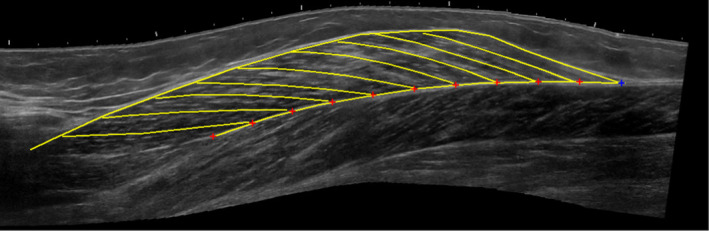
Processed panoramic ultrasound image of medial gastrocnemius with manually labeled superficial and deep aponeuroses and nine fascicles.

Ultrasound images were processed with a custom‐made python script that supported the operator (author Jule Heieis) to manually outline the architectural features. First, the operator identified the superficial and deep aponeuroses. Then they identified up to nine clearly visible fascicles, each with as many linear segments s as needed to replicate the aponeurosis' or fascicle's shape (Figure 2). Only fascicles that were visible over a wide range were included into analysis. Sections of the fascicle that were not visible were treated as straight lines.

**FIGURE 3:**
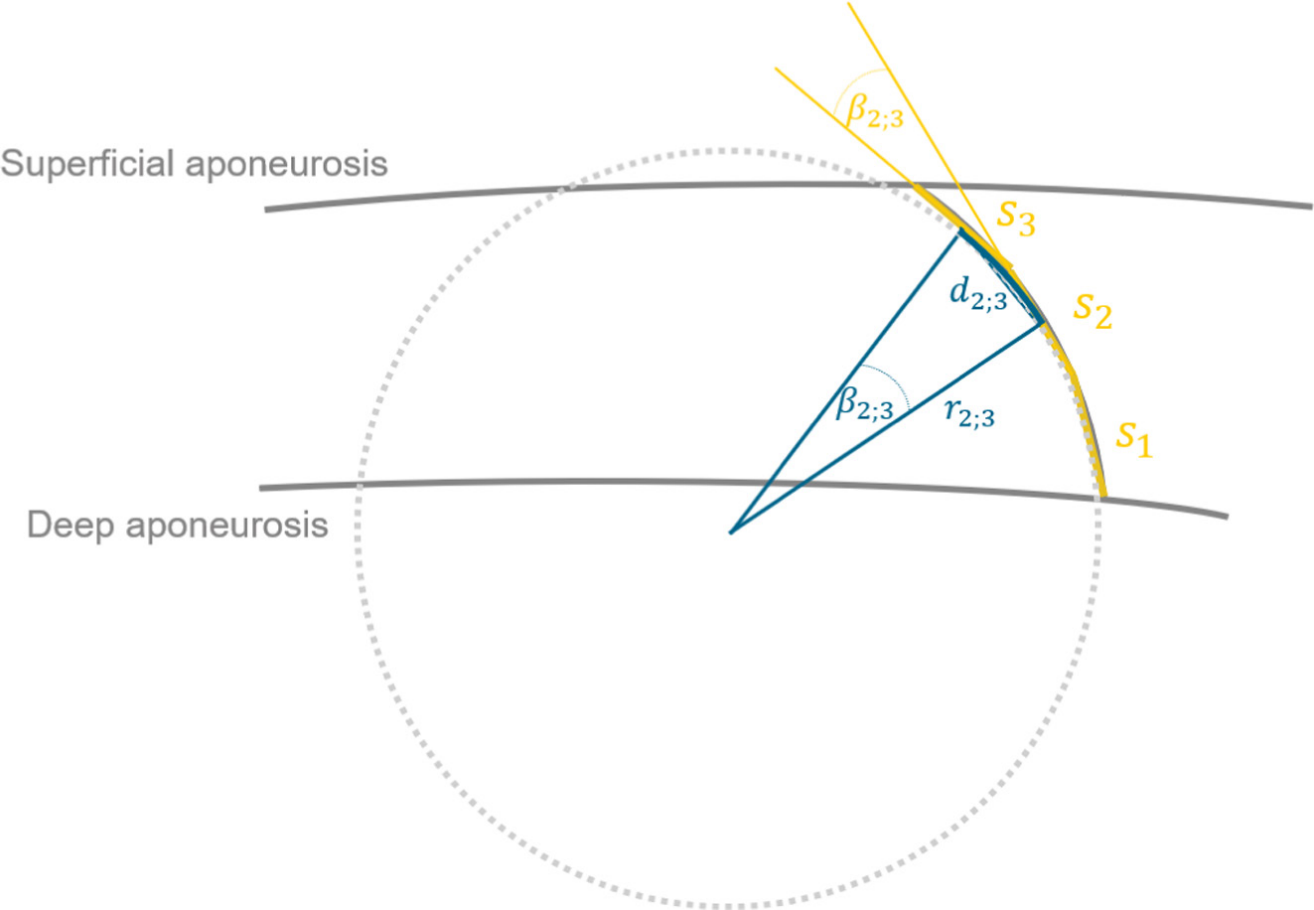
Segment curvature $c_{s,2;3}$ calculation based on circle section fitting. The fitted circle section is defined by the segments' $s_{2}$ and $s_{3}$ midpoints as circle points and their distance $d_{2;3}$ as circle chord. $c_{s,2;3}$ is calculated as inverse circle radius $r_{2;3}$ using Equation (1).

Fascicle length $l_{f}$ was defined as the sum of all segment lengths $l_{s}$ of the corresponding fascicle. Pennation angles were measured between the end segments of a fascicle and the deep aponeurosis segment they intersect with. The python script used for image processing calculated curvature between two neighboring fascicle segments by fitting a circle sector to both segments' midpoints as points of the circle and their connecting line d as circle chord (Figure 3). Segment curvature $c_{s}$ was then defined as the inverse radius *r* of the fitted circle with the formula for circle segment chords,

(1) $$c_{s}=\frac{1}{r}=\frac{2\sin\left(\frac{\beta }{2}\right)}{d}$$

where β is the supplementary angle to the angle between the segments (Figure 3). Curving with the concave side toward the deep aponeurosis was defined as positive, while the opposite was defined as negative curvature. After image processing all further data processing and analysis has been performed using the R software package (www.r‐project.org, version 4.1.2) within the R‐Studio environment (https://posit.co). The total curvature of a fascicle $c_{f}$ was calculated as the weighted mean of all n segment curvatures $c_{s}$ of the fascicle with the formula

(2) $$c_{f}=\frac{\sum_{i=1}^{n}w_{i}c_{s,i}}{\sum_{i=1}^{n}w_{i}}$$

with

(3) $$w_{i}=l_{s,i}+l_{s,i+1}$$

Finally, the mean values of the architectural parameters throughout the muscle were calculated by averaging them in two steps, first by calculating the mean for the proximal, central, and distal third of the muscle separately before secondly averaging the three means to correct for potentially imbalanced analysis of fascicles throughout the muscle. Mean fascicle strain was calculated as relative length change of mean fascicle length during contraction compared to rest. The intramuscular position of the fascicles was defined by their insertion point into the deep aponeurosis relative to the deep aponeurosis length.

The motion capture data were processed using the Vicon Nexus 1.8.5 software (Vicon Motion Systems Ltd). Gaps in marker position recording were filled using the “Fill” function provided by the software. Ankle and knee joint kinematics were analyzed for each frame in the participant's sagittal plane. Body segments were defined by one proximal and one distal marker each (Table 1). The body segment vectors of thigh, shank, and foot were then projected orthogonally onto the measurement volume's sagittal plane YZ, defined as Y to be aligned with the lateral edge of the IsoMed 2000 seat and Z to be oriented vertically. MTC lengths of the medial and lateral gastrocnemius could then be calculated with the joint angles using the formulas of Hawkins and Hull (1990) and were averaged over the whole phase of ultrasound imaging during constant contraction. To emphasize changes in MTC length it was transformed to MTC strain $\varepsilon_{\mathrm{MTC}}$ by calculating the relative length change compared to the MTC length in the middle of the five testing positions, namely 135° knee and 90° ankle angle.

Plantarflexion torque $M_{\mathrm{PF}}$ was filtered with a 20 Hz low‐pass filter, converted relative to the MVC and averaged over the time of image acquisition.

### 2.5 Statistical analyses

Study demographics have been statistically analyzed between groups by independent *t*‐tests for numerical data and chi‐squared test for categorical data.

Before statistical analysis ${\bar{l}}_{f}$ was reciprocally (*x* ^−1^) transformed to fulfill the rules of linearity for linear mixed models. Correlations between all parameters were investigated using the “rmcorr” R‐package for repeated measures correlation coefficients.

Mean curvature of all segmented fascicles within the medial gastrocnemius (${\bar{c}}_{f_{\mathrm{GM}}}$) was subjected to statistical analyses with four competing linear mixed models with, either (A) plantar flexion torque ($M_{\mathrm{PF}}$) and MTC strain ($\varepsilon_{\mathrm{MTC}}$), (B) mean pennation angle (${\bar{\alpha }}_{d}$), (C) mean fascicle length (${\bar{l}}_{f}$), or (D) mean fascicle strain (${\bar{\varepsilon }}_{f}$) as independent variables (respectively, models 1A, 1B, 1C, and 1D). The best fitted model, checked by lowest Akaike information criterion (AIC), was expanded for intermuscular (adding muscle type as fixed two‐level factor and interaction term with independent variable) and for intramuscular (adding intramuscular position as fixed effect) analysis as models 2 and 3. For potential age‐ and sex‐related effects, models 1, 2, and 3 were repeated once with age group and once with sex, both by incorporating these factors in the models as fixed two‐level factor and interaction term with the independent variable. To quantify the predictive capacities of the models, their marginal and conditional *R* ^2^ values have been calculated. Data are given as mean, standard deviation (SD), and median. The significance threshold is set to 5%.

## 3 RESULTS

### 3.1 Participants

The 12 study participants are divided into 6 “young” (18–30 years old) and 6 “old” (60–70 years old) participants. Our sample also includes equal ratios of “male” and “female” participants. Participants' anthropometric data are given in Table 2.

**TABLE 2:** Anthropometric data of the test participants.

Variable	Old				Young				Test
	*N*	Mean	SD	Median	*N*	Mean	SD	Median	
Age (years)	6	63.7	1.7	64	6	24	4.4	25	*t* = 20.4^‡^
Sex	6				6				*X* ^2^ = 0
Female	3	50%			3	50%			
Male	3	50%			3	50%			
Height (cm)	6	173	5.7	172.5	6	172.5	9.6	172.5	*t* = 0.1
Weight (kg)	6	70.8	11.3	72.5	6	66.4	8	69	*t* = 0.8

Statistical significance markers: ^‡^ *p* < 0.001.

### 3.2 Mean fascicle curvature in medial gastrocnemius muscles

Repeated measures correlation coefficients for all analyzed parameters are provided in Table 3. Correlation coefficients larger than 0.8 are found between the mean deep pennation angle ${\bar{\alpha }}_{d}$ and mean fascicle length ${{\bar{l}}_{f}}^{-1}$ (0.94), as well as between the relative plantar flexion torque *M*_PF_ and mean fascicle strain ${\bar{\varepsilon }}_{f}$ (−0.8). According to the low correlation between relative plantar flexion torque *M*_PF_ and MTC strain $\varepsilon_{\mathrm{MTC}}$ (−0.23), we do not expect collinearity within model 1A.

**TABLE 3:** Repeated measures correlation coefficients between all analyzed parameters.

Variable	${\bar{c}}_{f_{\mathrm{GM}}}$	*M*_PF_	$\varepsilon_{\mathrm{MTC}}$	${\bar{\alpha }}_{d}$	${{\bar{l}}_{f}}^{-1}$	${\bar{\varepsilon }}_{f}$
${\bar{c}}_{f_{\mathrm{GM}}}$	1					
*M*_PF_	0.4^‡^	1				
$\varepsilon_{\mathrm{MTC}}$	−0.25^‡^	−0.23^‡^	1			
${\bar{\alpha }}_{d}$	0.63^‡^	0.7^‡^	−0.58^‡^	1		
${{\bar{l}}_{f}}^{-1}$	0.49^‡^	0.65^‡^	−0.69^‡^	0.94^‡^	1	
${\bar{\varepsilon }}_{f}$	−0.38^‡^	−0.8^‡^	0.08	−0.71^‡^	−0.64^‡^	1

Abbreviations: ${\bar{c}}_{f_{\mathrm{GM}}}$, mean fascicle curvature; *M*_PF_, relative plantar flexion torque; $\varepsilon_{\mathrm{MTC}}$, muscle–tendon complex strain; ${\bar{\alpha }}_{d}$, mean deep pennation angle; ${\bar{l}}_{f}$, mean fascicle length; ${\bar{\varepsilon }}_{f}$, mean fascicle strain.

Statistical significance markers: ^‡^ *p* < 0.001.

Model 1A reveals that mean fascicle curvature is negatively oriented at rest. With increasing *M*_PF_, ${\bar{c}}_{f_{\mathrm{GM}}}$ shifts positively by +0.05 m^−1^ for every 1% MVC increase (p=0.004), thus suggesting a positive shift of curvature by +5 m^−1^ at 100% MVC. The random slope estimates of $M_{\mathrm{PF}}$ were consistently positive for all participants, but varied noticeably in their magnitude (range of 0.008–0.124 m^−1^ per % MVC). For $\varepsilon_{\mathrm{MTC}}$, the random slope estimates varied between participants in magnitude and direction (range of −0.873 to 0.378 m^−1^ per %), hence $\varepsilon_{\mathrm{MTC}}$ had no significant impact on ${\bar{c}}_{f_{\mathrm{GM}}}$ (p=0.375).

**FIGURE 4:**
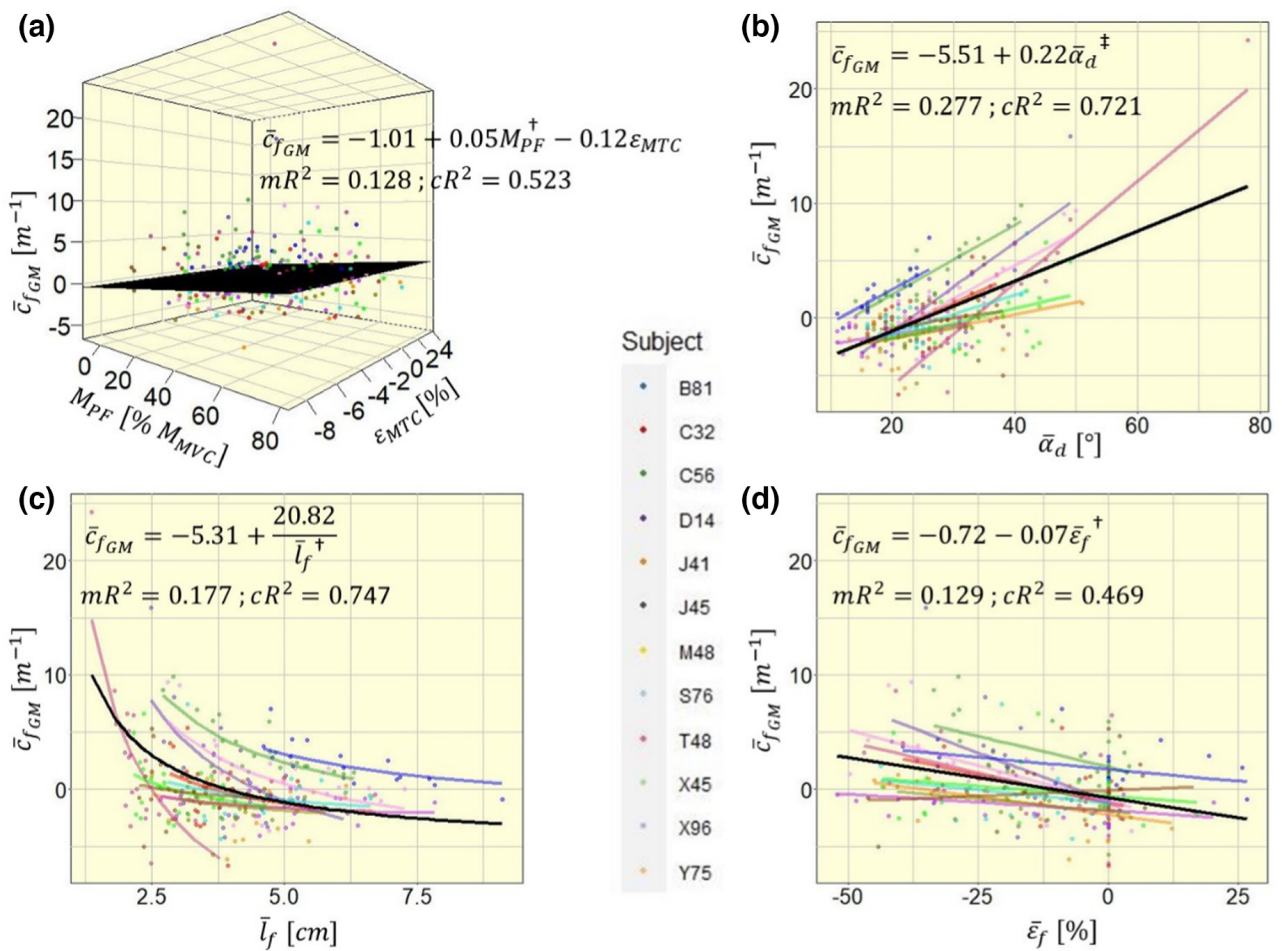
Four competing models to explain mean fascicle curvature in the medial gastrocnemius $\;{\bar{c}}_{f_{\mathrm{GM}}}$ with different independent variables: (a) with muscle–tendon complex strain $\varepsilon_{\mathrm{MTC}}$ and contraction level $M_{\mathrm{PF}}$; (b) with mean deep pennation angle ${\bar{\alpha }}_{d}$; (c) with mean fascicle length ${\bar{l}}_{f}$; (d) with mean fascicle strain ${\bar{\varepsilon }}_{f}$. Model fit equations are given within the plot, visualized via a black plane for a and black lines for b–d. For simplicity, model estimates within the equations are displayed without units. mR^2^, marginal *R* ^2^; cR^2^, conditional *R* ^2^. Significance markers: ^†^ *p* < 0.01; ^‡^ *p* < 0.001.

Model 1B estimated a significant negative to positive shift in ${\bar{c}}_{f_{\mathrm{GM}}}$ of +0.22 m^−1^ with every degree of ${\bar{\alpha }}_{d}$ (Figure 4b; p<0.001). Correspondingly, ${\bar{c}}_{f_{\mathrm{GM}}}$ shifts negatively with increasing ${\bar{l}}_{f}$ (model 1C; 20.82 m^−1^ per cm^−1^; Figure 4c; p=0.003), ${\bar{c}}_{f_{\mathrm{GM}}}$ also shifts negatively with increasing ${\bar{\varepsilon }}_{f}$ (model 1D; 0.07 m^−1^ per %; Figure 4d; p=0.004). In models 1B and 1C, all random slope estimates were consistent in their direction for individual participants (Figure 4b,c). Participant Y75 was the only participant with positive slope estimate in model 1D, while all other participants had negative slope estimates (Figure 4d).

Model 1B was the best model to explain model variance with its fixed effect, quantified by the highest marginal *R* ^2^ (0.128, 0.277, 0.177, and 0.129, respectively, for models 1A, 1B, 1C, and 1D), while model 1C had best variance explanation by fixed and random effects with highest conditional *R* ^2^ (0.523, 0.721, 0.747, and 0.469, respectively, for models 1A, 1B, 1C, and 1D). Comparing the four models' AIC values we found the lowest AIC and consequently best fit with model 1B (1339, 1263, 1338, and 1301, respectively, for models 1A, 1B, 1C, and 1D). Hence, the following analyses were performed using ${\bar{\alpha }}_{d}$ as main explanatory variable.

### 3.3 Inter‐muscular differences in mean fascicle curvature

**FIGURE 5:**
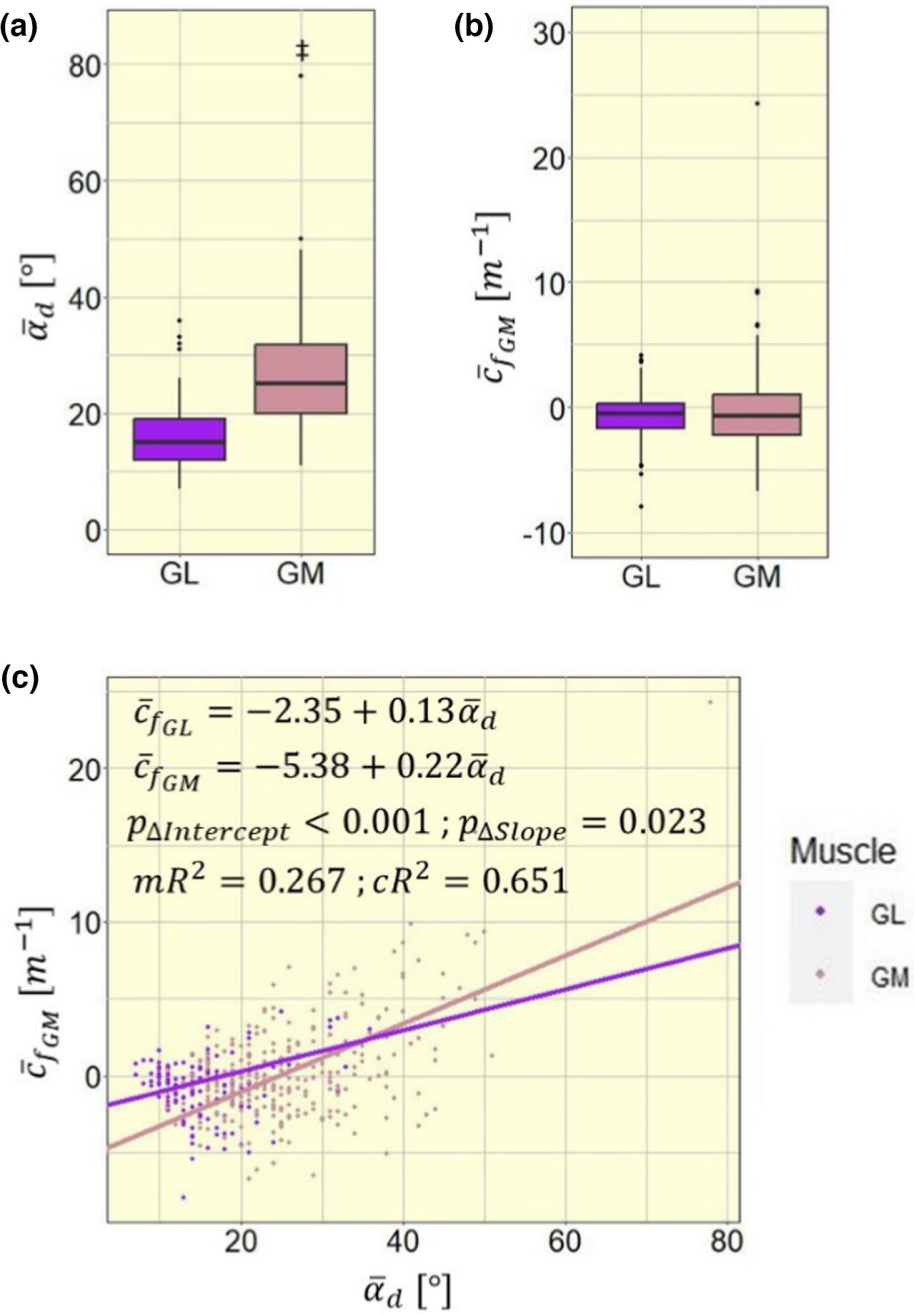
Intermuscular differences (a) in mean fascicle curvature (${\bar{c}}_{f}$), (b) in mean pennation angle (${\bar{\alpha }}_{d}$), and (c) in model intercept and slope between medial and lateral gastrocnemius (GM and GL); mR^2^, marginal *R* ^2^; cR^2^, conditional *R* ^2^. Significance markers: ^‡^ *p* < 0.001.

The average deep pennation angle ${\bar{\alpha }}_{d}$ differed between medial and lateral gastrocnemius (Figure 5a; *t* = 15.452; *p* < 0.001), while the average fascicle curvature ${\bar{c}}_{f}$ did not show significant differences between the muscles (Figure 5b; *t* = 1.7533; *p* = 0.082). The muscle was included into the model as a two‐level fixed factor Muscle, which is equal to 1 for the medial and to 0 for the lateral gastrocnemius, and as an interaction term with ${\bar{\alpha }}_{d}$, to investigate differences between muscles in regards to model intercept and slope. This leads to the following model equation:

(4) $$\begin{array}{c} {\bar{c}}_{f}=-2.35m^{-1}+0.13\frac{m^{-1}}{{}^{\circ}}*{\bar{\alpha }}_{d}-3.04m^{-1}*\text{Muscle}\\ \;+0.09\frac{m^{-1}}{{}^{\circ}}*{\bar{\alpha }}_{d}*\text{Muscle}, \end{array}$$

which reduces to

(5) $${\bar{c}}_{f_{\mathrm{GM}}}=-5.38m^{-1}+0.22\frac{m^{-1}}{{}^{\circ}}*{\bar{\alpha }}_{d}$$

and

(6) $${\bar{c}}_{f_{\mathrm{GL}}}=-2.35m^{-1}+0.13\frac{m^{-1}}{{}^{\circ}}*{\bar{\alpha }}_{d}$$

for the medial and lateral gastrocnemius, respectively. The fixed factor Muscle and the interaction term ${\bar{\alpha }}_{d}*\text{Muscle}$ both had significant impact on ${\bar{c}}_{f}$ (respectively, p<0.001 and p=0.023), showing significant differences in model intercept and slope between the muscles (Figure 5c).

### 3.4 Intramuscular differences in fascicle curvature

**FIGURE 6:**
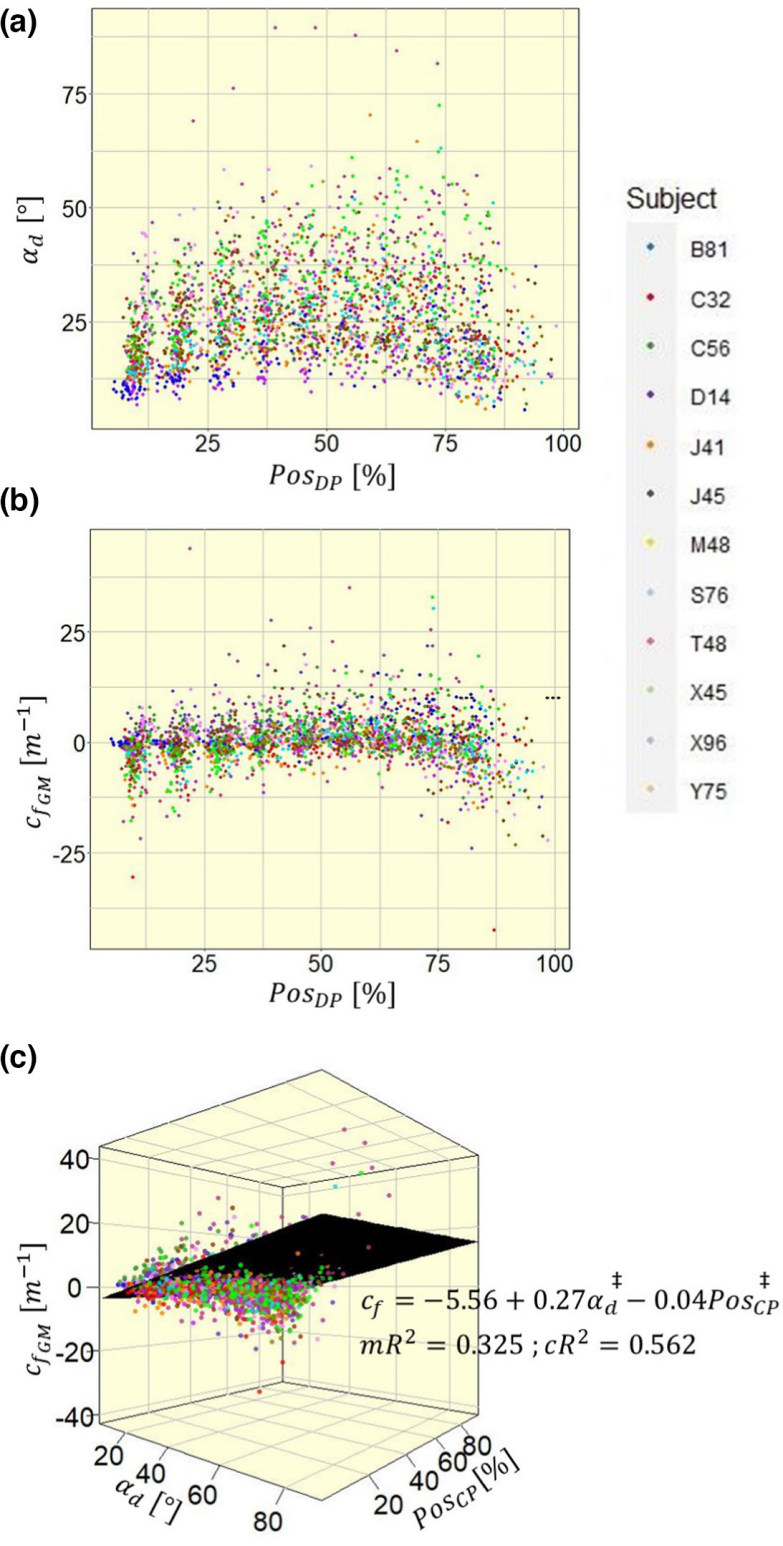
Distribution of (a) pennation angle ($\alpha_{d}$) and (b) fascicle curvature ($c_{f}$) from distal to proximal intramuscular position (${\mathrm{Pos}}_{\mathrm{DP}}$), in (c) combined into a linear mixed model with intramuscular position defined from central to peripheral (${\mathrm{Pos}}_{\mathrm{CP}}$) to create linearity. mR^2^, marginal *R* ^2^; cR^2^, conditional *R* ^2^. Significance markers: ^‡^ *p* < 0.001.

$c_{f}$ and $\alpha_{d}$ both show similarly shaped distributions throughout the muscle (Figure 6a,b). Both parameters exhibit larger values in the central muscle region than in the peripheral regions. To model linear relationships, the intramuscular position ${\mathrm{Pos}}_{\mathrm{CP}}$ was defined from central to peripheral. Besides the significant influence of $\alpha_{d}$, with an estimate of 0.27 m^−1^ per degree (Figure 6c; p<0.001), ${\mathrm{Pos}}_{\mathrm{CP}}$ also has a significant impact on $c_{f}$, with an estimated increase of 3.64 m^−1^ from the MTJs to the muscle center (Figure 6c; p<0.001).

### 3.5 Age‐ and sex‐related effects

No model showed significant impact of age on fascicles' curvature ${\bar{c}}_{f}$. The intramuscular differences, however, differed significantly between sexes. In male participants, $c_{f}$ decreased by 2.2 m^−1^ from central toward peripheral muscle regions, while it decreased by 5 m^−1^ in female participants (p<0.001).

## 4 DISCUSSION

Our findings suggest that positive and negative curvature appear under different circumstances. While negative curvature can mainly be seen in passive muscles, positive curvature emerges during activity. Contraction leads to a decrease in muscle length, with the associated architectural hallmarks of increased pennation and fascicle shortening. Hypotheses regarding the influence of both these parameters will be discussed in the following.

### 4.1 The relationship between fascicle length and fascicle curvature

Our data shows a hyperbolic rather than linear relationship between fascicle length and curvature, with curvature increasing substantially at short fascicle lengths (Figure 4c). Short fascicles fall “slack,” which results in lack of passive tension and low stiffness of the fascicles (Herbert et al., 2011; Hug et al., 2013). In slack or non‐stretched fascicles, the longitudinal tensile forces of passive elements cease, so that laterally acting stresses or pressures could become relatively more effective than fascicle‐aligned stresses. This change in predominance of laterally acting stresses over fascicle‐aligned stresses could thus result in the orthogonal displacement of fascicles (i.e., fascicles curving). Additionally, fascicles of the same muscle fall slack at different muscle length, which could be a reason for intramuscular differences in fascicle curvature. This hypothesis relies on the presence of laterally acting forces within the muscle. In non‐human species, indications of non‐myotendinous force transmission have been found (Huijing et al., 1998; Monti et al., 1999; Passerieux et al., 2006). In humans this evidence is still missing. However, the human perimysium and endomysium constitute three‐dimensional structures that, as per its anatomy, can transmit stresses between neighboring contractile elements at almost all hierarchical levels of muscle structure, which could potentially transmit stresses laterally.

### 4.2 The relationship between pennation angle and fascicle curvature

Other than fascicle length, pennation angle and fascicle curvature correlate linearly, with a significant positive shift of fascicle curvature with increasing pennation angle. With increasing pennation angle the fraction of fiber force oriented perpendicular to the muscle's axis of force production increases, which causes an increase in hydrostatic pressure (Styf et al., 1995). Correspondingly, not only pennation angle increases during contraction, but also the intramuscular pressure (Petrofsky & Hendershot, 1984; Sejersted et al., 1984). So, it can be hypothesized, that fascicle curvature not only correlates with pennation angle, but also with intramuscular pressure. This assumption is supported by the intramuscular distributions of fascicle curvature and intramuscular pressure. It has been found in canine gastrocnemius‐plantaris muscles that the maximum intramuscular pressure is highest in central and deep regions of the muscle (Ameredes & Provenzano, 1997). This central‐peripheral pattern could also be seen in the intramuscular distribution of fascicle curvature in our experimental data of the medial gastrocnemius, where positive curvature appears in the central muscle, while negative curvature appears in the peripheral muscle (see Figure 6b). It contrasts with results from Muramatsu et al., who found no intramuscular curvature pattern (Muramatsu et al., 2002), and (Namburete & Wakeling, 2012), who observe a distal‐proximal pattern (Namburete & Wakeling, 2012). Not only internal pressures within the muscle, but also pressures between adjacent muscles could influence muscle and fascicle shape. It has been shown in canine gastrocnemius and plantaris muscles that intermuscular pressures rise linearly with contraction (Reinhardt et al., 2016). Interestingly the increase in pressure correlates with contraction and not with muscle–tendon complex length, similar to fascicle curvature in our experiments. An influence of muscle interactions on fascicle curvature is conceivable.

In general, the influences of fascicle length and pennation angle should not be interpreted independently, because of their very high correlation (Table 3). The resultant hypotheses presented here to rationalize the influence of intramuscular connective tissue, fascicle curvature and intramuscular pressure, should therefore not be seen as contradicting each other. A combination of phenomena is likely to explain fascicle curving.

The systematic link between fascicle curvature, pennation angle, and fascicle length has implications. First, it defies the simplistic view of fascicles acting merely as “stiffening cables” that exert pulling forces between superficial and deep aponeuroses during contraction. Rather, it seems that contraction‐related pressures and/or stresses that are unaligned with the fascicles affect the stress–strain behavior within the muscle. As a corollary of that notion, the mechanical properties and actions of intramuscular connective tissue come to mind. The internal stress–strain behavior of muscle has potential to affect stiffness, elasticity, efficiency, and other mechanical traits of transmission between sarcomeres and tendon. We have demonstrated in this study that the emergence of fascicle curvature differs not only between different muscles, but that it could also be subject to changes with age or sex. Thus, the observed sex‐related effect on curvature distribution within the muscle leads us to speculate that sex hormones influence the underlying mechanics of fascicle curving. It is known that estrogen stimulates the collagen synthesis (Hansen et al., 2009), which correlates with findings of reduced response to mechanical loading (Magnusson et al., 2007), lower stiffness, and greater tendon strain in women (Kubo et al., 2003). Tendon and muscle connective tissue mechanics might thereby have an impact on the intramuscular mechanics and fascicle curvature. Although it would be precocious to draw definite conclusions on sex‐related differences in muscle's stress–strain behavior, our results do provide supporting evidence for future studies in that direction.

Finally, this study failed to show any age‐related effects. However, if evidence for the role of intramuscular connective tissue in the emergence of fascicle curvature can be found in future studies, age‐related effects might still be of interest, because of changes in intramuscular connective tissue content and composition with age (Alnaqeeb et al., 1984).

### 4.3 Limitations

For analyses regarding sex and age the sample size is too small to draw definite conclusions. Additionally, as with every assessment of muscle architecture by 2D ultrasound, there is a risk of measurement error due to 3D effects and motion that cannot be controlled for with 2D imaging. By analyzing only mostly visible fascicles and collecting the images at the medio‐lateral centerline we are confident that this measurement error could be minimized.

Participants only performed one MVC trial per position. Additionally, the duration and amount of contraction could potentially lead to fatigue effects. To account for this, the order of positions has been randomized.

## 5 CONCLUSION

Using a systematic examination protocol and panoramic ultrasound imaging, we propose a detailed description of fascicle curvature as a function of muscle–tendon complex length and contraction level. Our results confirm the influence of contraction level on fascicle curvature (Muramatsu et al., 2002; Namburete & Wakeling, 2012; Rana et al., 2014). Strong correlations have also been found between fascicle curvature and pennation angle, and fascicle curvature and the inverse fascicle length. The influence of pennation angle on fascicle curvature differs significantly between medial and lateral gastrocnemius. Regarding intramuscular variations, we found a linear relation to curvature from central to peripheral, with significant differences according to sex.

Based on our findings, parameters that could be investigated in connection with fascicle curving are the stiffnesses of muscle and tendon tissue and tendon mechanics. These parameters should especially be investigated in regards to age‐, sex‐, and sex hormone‐related differences. Future research should especially pay attention to the link between intramuscular pressure and fascicle curvature, because both parameters show similar correlations to contraction and pennation angle and similar intramuscular distributions.

## AUTHOR CONTRIBUTIONS

*Conception of study*: Jörn Rittweger, Olfa D'Angelo, Uwe Mittag, Thomas Voigtmann, Andreas Meyer. *Implementation of study*: Jörn Rittweger, Jule Heieis, Jonas Böcker. *Data collection*: Jule Heieis, Jonas Böcker, Olfa D'Angelo. *Data processing*: Jule Heieis, Uwe Mittag, Thomas Voigtmann, Olfa D'Angelo, Kirsten Albracht. *Statistical analyses*: Jule Heieis, Jörn Rittweger. *Interpretation of results*: all authors. *Drafting manuscript*: Jule Heieis, Jörn Rittweger. *Editing and approving manuscript*: all authors.

## FUNDING INFORMATION

The study has been conducted using only internal funding of the Institute of Aerospace Medicine and has been approved by the North Rhine Medical Association's ethics committee.

## CONFLICT OF INTEREST STATEMENT

The authors declare no conflicts of interest. The results of the study are presented clearly, honestly, and without fabrication, falsification, or inappropriate data manipulation.

## ETHICAL STATEMENT

The participants gave their informed consent prior to their inclusion in the study.

## Data Availability

The data that support the findings of this study are openly available in “figshare” at https://doi.org/10.6084/m9.figshare.21856818.v2.
